# Early oral feeding is safe and useful after rectosigmoid resection with anastomosis during cytoreductive surgery for primary ovarian cancer

**DOI:** 10.1186/s12957-021-02186-6

**Published:** 2021-03-15

**Authors:** Kazuyoshi Kato, Kohei Omatsu, Sanshiro Okamoto, Maki Matoda, Hidetaka Nomura, Terumi Tanigawa, Yoichi Aoki, Mayu Yunokawa, Hiroyuki Kanao

**Affiliations:** 1 Department of Gynecology, Cancer Institute Hospital, 3-8-31 Ariake, Koutou-ku, Tokyo, 135-8550 Japan; 2 Present address: Department of Obstetrics and Gynecology, Tokyo Medical University, 6-7-1 Nishishinjuku, Shinjuku-ku, Tokyo, 160-0023 Japan

**Keywords:** Ovarian cancer, Rectosigmoid resection, Early oral feeding, Postoperative morbidity, Postoperative hospital stay

## Abstract

**Background:**

The aim of this study was to investigate the safety and clinical usefulness of early oral feeding (EOF) after rectosigmoid resection with anastomosis for the treatment of primary ovarian cancer.

**Methods:**

We performed a retrospective review of all consecutive patients who had undergone rectosigmoid resection with anastomosis for primary ovarian, tubal, or peritoneal cancer between April 2012 and March 2019 in a single institution. Patient-related, disease-related, and surgery-related data including the incidence of anastomotic leakage and postoperative hospital stay were collected. EOF was introduced as a postoperative oral feeding protocol in September 2016. Before the introduction of EOF, conventional oral feeding (COF) had been used.

**Results:**

Two hundred and one patients who underwent rectosigmoid resection with anastomosis, comprised of 95 patients in the COF group and 106 patients in the EOF group, were included in this study. The median number of postoperative days until the start of diet intake was 5 (range 2–8) in the COF group and 2 (range 2–8) in the EOF group (*P* < 0.001). Postoperative morbidity was equivalent between the groups. The incidence of anastomotic leakage was similar (1%) in both groups. The median length of the postoperative hospital stay was reduced by 6 days for the EOF group: 17 (range 9–67) days for the COF group versus 11 (8–49) days for the EOF group (*P* < 0.001).

**Conclusion:**

EOF provides a significant reduction in the length of the postoperative hospital stay without an increased complication risk after rectosigmoid resection with anastomosis as a part of cytoreductive surgery for primary ovarian cancer.

## Background

A maximal surgical effort to obtain a macroscopic complete resection has been confirmed to improve survival among patients with advanced ovarian cancer [1–3]. An en bloc resection of the rectosigmoid together with the uterus, tubes/ovaries, and the pelvic tumors, also known as modified posterior pelvic exenteration or radical oophorectomy, is one of the most frequently performed types of surgical procedures during cytoreductive surgery for advanced ovarian cancer [4–7]. Anastomotic leakage remains a life-threatening potential complication of rectosigmoid resections despite advances in surgical techniques and medical instrumentation [8, 9]. As cytoreductive surgery aimed at complete resection requires the removal of multiple organs and the resection of extensive amounts of the peritoneum, it is usually complicated and invasive. Rectosigmoid resection with anastomosis is regarded as the most complicated surgical procedure among the various procedures that might be required during cytoreductive surgery for advanced ovarian cancer, including diaphragmatic resection, splenectomy, and hepatectomy as well as large and small bowel resections [10].

Enhanced recovery after surgery (ERAS) has been developed to accelerate recovery by attenuating the stress response. Previous studies have demonstrated that ERAS perioperative care affects neural, metabolic, and other organ functions beneficially [11, 12]. Most data supporting ERAS programs have come from colorectal surgery. The key components for ERAS in colorectal surgery are as follows: a thorough preoperative patient history, thoracic epidural anesthesia during open (but not laparoscopic) colonic surgery, the avoidance of fluid overload and hypovolemia, no use of a nasogastric tube, and early postoperative oral feeding (EOF) and mobilization [12–15]. Consequently, this multimodal approach has been shown to shorten the length of the hospital stay, reduce the surgical stress response, decrease morbidity, and expedite recovery. As part of ERAS programs, there has recently been a move toward EOF regimens postoperatively. The benefits of EOF include a faster recovery of bowel function and lower rates of infectious complications including anastomotic leakage [14]. ERAS programs were introduced to the field of gynecologic oncology. Then, some studies have demonstrated that increased ERAS guideline compliance is associated with a decrease in the length of the hospital stay across all patients and a lower risk of complications [16–18].

As high-complexity surgery is more frequently performed for primary ovarian cancer surgery than for colorectal or ordinary gynecologic surgery, whether EOF is also safe for ovarian cancer patients who undergo rectosigmoid resection with anastomosis as a part of cytoreductive surgery remains unknown. We evaluated the impact of EOF on postoperative outcomes, including postoperative hospital stay, following rectosigmoid resection.

## Methods

### Patient selection

All consecutive patients who underwent rectosigmoid resection with anastomosis as part of cytoreductive surgery for primary ovarian, tubal, or peritoneal cancer at the Cancer Institute Hospital, Japan, between April 2012 and March 2019 were evaluated for inclusion in this study. In April 2012, well-trained gynecologic oncologists started to perform cytoreductive surgery, including small and large bowel resection with anastomoses, stoma creation, diaphragm stripping/resection with liver mobilization, and splenectomy with distal pancreatectomy, at our institution. The patients’ medical records were reviewed, and patient-related, disease-related, and surgery-related data were retrieved. These data included the patient’s age, performance status, medical history, primary site of disease, clinical stage, timing of surgery (up-front surgery or interval debulking surgery), preoperative laboratory values including the serum levels of hemoglobin and albumin, surgical procedures, residual disease, operative time, estimated blood loss, intraoperative blood transfusion, final histopathologic results, diet resumption, length of postoperative hospital stay, duration from surgery until the start of postoperative chemotherapy, and morbidity within 60 days after surgery. The complexity of the surgical procedures performed was stratified in accordance with the previously advocated surgical complexity score (SCS) system into 3 groups (low, moderate, and high) [10]. Perioperative complications were registered and classified according to the Clavien–Dindo classification [19]. Preoperative bowel preparation was identical throughout the study period. Patients were given 180 mL of magnesium citrate on the day before surgery. Oral antibiotic bowel preparation was not used, and prophylactic IV antibiotic therapy with cephalosporin was administered immediately before and during surgery in the current series. Since the middle of this study period, we have routinely utilized a transanal drainage tube (TDT) after rectosigmoid resection with anastomosis for patients with ovarian cancer to prevent anastomotic leakage [20]. This study protocol was approved by the Institutional Review Board of the Cancer Institute Hospital.

### Timing of postoperative oral feeding and discharge

EOF after rectosigmoid resection with anastomosis was introduced at our hospital in September 2016. Patients in the EOF group began to drink water and/or tea on postoperative day (POD) 1. If these liquids were well tolerated, they advanced to a semisolid diet on POD 2 and to a regular diet within the next 2 to 3 days. Before the introduction of EOF, postoperative oral feeding had been managed using a conventional methodology. Patients in the conventional oral feeding (COF) group were managed with nothing by mouth until postoperative flatus was noted. Thereafter, they were allowed to drink water. If the water was tolerated, a liquid diet was given. They then advanced to a regular diet within the next 5 to 7 days. The durations of oral feeding prohibition in the patients who underwent a splenectomy combined with a distal pancreatectomy to achieve complete cytoreduction depended on the grade of pancreatic fistula. We placed a peripancreatic drain into the left subdiaphragmatic space in all the patients who underwent a splenectomy with a distal pancreatectomy for the diagnosis and management of pancreatic fistula [21]. Diet intake was started after the pancreatic fistula was confirmed to be resolved.

The criteria for discharge were as follows: whether the patient could eat a regular diet, walk for herself, make a living by herself, and get ready for postoperative chemotherapy. Gynecologic oncologists in our institution checked her conditions and talked the timing of discharge from hospital with her and her family.

### Statistical analyses

Statistical analyses were performed using the Mann-Whitney *U* test for continuous variables and the chi-square test or the Fisher exact test for categorical variables with SPSS version 18.0. A value of *P* < 0.05 was considered statistically significant.

## Results

During this study period, 202 patients underwent rectosigmoid resection as part of cytoreductive surgery for primary ovarian, tubal, or peritoneal cancer at the time of initial therapy. One patient who underwent a Hartmann’s procedure without colorectal anastomosis was excluded from this study. Finally, 201 patients who underwent rectosigmoid resection with anastomosis were evaluated in the current series.

**Fig. 1:**
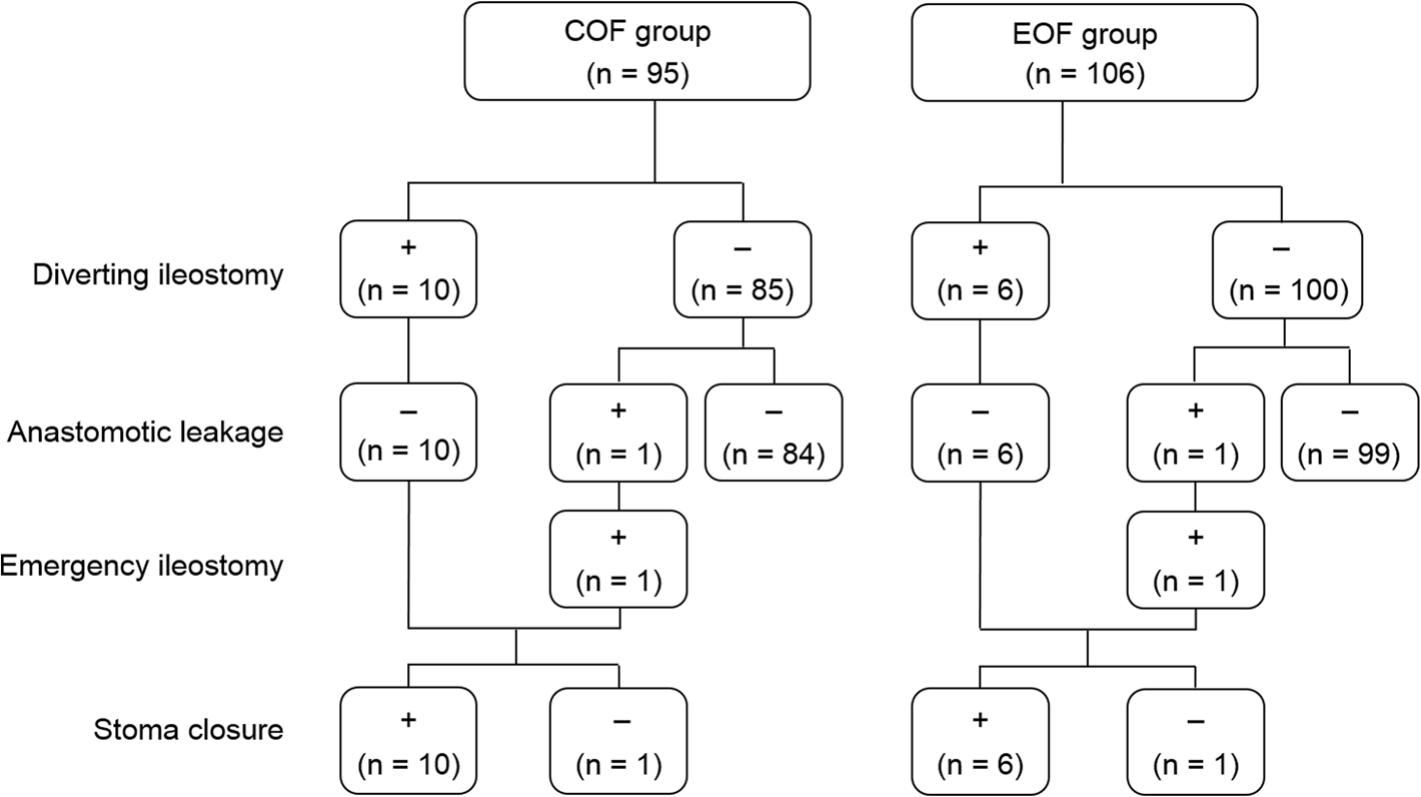
The distribution of the patients who underwent modified posterior pelvic exenteration for the treatment of primary ovarian cancer. A diverting ileostomy was created in 10 patients (11%) in the conventional oral feeding (COF) group (*n* = 95) and 6 patients (6%) in the early oral feeding (EOF) group (*n* = 106). Two patients developed anastomotic leakage and required an emergency ileostomy. Stoma closure was performed in 16 of the 18 patients at 1–3 months after the completion of the postoperative chemotherapy

The patients who underwent surgery between April 2012 and August 2016 were assigned to the COF group (*n* = 95). The patients who underwent surgery between September 2016 and March 2019 were assigned to the EOF group (*n* = 106). The baseline characteristics of both groups were similar (Table 1). No differences were found between the two groups according to the surgical variables (Table 2). In addition to the rectosigmoid resection, 21 patients (22%) in the COF group and 26 patients (25%) in the EOF group underwent one or more intestinal resection(s). Fifty-seven patients (60%) in the COF group and 60 patients (57%) in the EOF group underwent upper abdominal surgery including diaphragmatic stripping/resection and splenectomy with distal pancreatectomy. A diverting ileostomy was created in 10 patients (11%) in the COF group and 6 patients (6%) in the EOF group (Fig. 1). The creation of the diverting ileostomy during cytoreductive surgery was determined by the gynecologic oncologists according to the perceived risk of anastomotic leakage in a case-by-case manner. The reasons for the stoma creation were as follows: (1) the condition that a rescue stoma had been already created before cytoreductive surgery as the management of colorectal obstruction in 3 patients; (2) intestinal edema because of serious loss of blood and a large quantity of transfused blood in 8 patients; (3) a very low colorectal anastomosis at 1.5–2 cm from the anal verge in 2 patients; (4) the occurrence of a fissure on the colonic wall during colorectal anastomosis using the circular stapling device in 1 patient; and (5) the presence of poorly controlled diabetes mellitus in 2 patients.

**Table 1:** Patient and tumor characteristics

	COF group (*n*=95)	EOF group (*n*=106)
Age, median (range), (years)	62 (30–82)	60 (33–80)
BMI, median (range), (kg/m^2^)	20.9 (13.8–33.0)	20.4 (14.0–32.6)
Presence of comorbidities, *n* (%)		
Diabetes mellitus	7 (7)	8 (7)
Hypertension	22 (23)	22 (20)
ASA score, *n* (%)		
1	33 (35)	30 (28)
2	31 (33)	37 (35)
3	30 (32)	37 (35)
4	1 (1)	2 (2)
Preoperative Hb, median (range), (g/dL)	11.1 (8.2–15.0)	11.6 (8.6–15.0)
Preoperative Alb, median (range), (g/dL)	4.0 (1.9–5.0)	4.0 (2.0–4.7)
Diagnosis, *n* (%)		
Ovarian cancer	86 (91)	94 (89)
Tubal cancer	4 (4)	5 (5)
Peritoneal cancer	5 (5)	7 (7)
Clinical stage, *n* (%)		
I	4 (4)	6 (6)
IIB	12 (13)	11 (10)
IIIA	5 (5)	4 (4)
IIIB	7 (7)	7 (7)
IIIC	44 (46)	48 (45)
IVA	1 (1)	4 (4)
IVB	22 (23)	26 (25)
Histology, *n* (%)		
Serous	65 (68)	71 (67)
Clear cell	12 (13)	19 (18)
Endometrioid	11 (12)	12 (11)
Mucinous	3 (3)	1 (1)
Others	4 (4)	3 (3)

*COF* conventional oral feeding, *EOF* early oral feeding, *BMI* body mass index, *ASA* American Society of Anesthesiologists, *Hb* hemoglobin, *Alb* albumin

**Table 2:** Surgical variables and postoperative complications

	COF group (*n*=95)	EOF group (*n*=106)	*P* value
Timing of cytoreductive surgery			
Up-front, *n* (%)	48 (51)	53 (50)	0.941
Interval, *n* (%)	47 (49)	53 (50)	
Surgical complexity score			
Intermediate, *n* (%)	41 (43)	40 (38)	0.434
High, *n* (%)	54 (57)	66 (62)	
Surgical procedures performed in addition to MPPE			
Splenectomy + distal pancreatectomy	3 (3)^b^	6 (6)^c^	0.608
Small and/or large bowel resection(s)	21 (22)^b^	26 (25)^c^	
Residual tumor after surgery, *n* (%)			
0	82 (86)	99 (93)	0.242
0<, < 1 cm	8 (8)	4 (4)	
≥ 1 cm	5 (5)	3 (3)	
Operative time, median (range), (min)	411 (222–689)	389 (196–719)	0.125
EBL, median (range), (mL)	840 (100–5600)	780 (130–6100)	0.519
Intraoperative transfusion of packed RBC, *n* (%)			
0	61 (64)	75 (70)	0.449
1–2 units	9 (9)	11 (10)	
>2 units	25 (26)	20 (19)	
Postoperative complications, *n* (%)^a^			
Grade 1–3a	20 (21)	18 (17)	0.759
Grade 3b–4	4 (4)	5 (5)	
Postoperative hospital stay, median (range), (days)	17 (9–67)	11 (8–49)	< 0.001
Duration from surgery to start of postoperative chemotherapy, median (range), (days)	35 (15–90)^d^	33 (12–77)^e^	0.080

*MPPE* modified posterior pelvic exenteration, *EBL* estimated blood loss, *RBC* red blood cells

^a^According to the Clavien–Dindo classification [15]

^b^The 1 patient underwent both bowel resections and splenectomy with distal pancreatectomy

^c^The 2 patients underwent both bowel resections and splenectomy with distal pancreatectomy

^d^The 3 patients who did not receive postoperative chemotherapy are excluded

^e^The 5 patients who did not receive postoperative chemotherapy are excluded

Immediately after surgery, none of the patients required the insertion of a nasogastric tube. Postoperative total parenteral nutrition was not used for any of them. After TDT placement was introduced at our institution, we routinely placed a TDT after rectosigmoid resection with anastomosis. Drainage of watery stool from the proximal side of the anastomosis through the TDT was noted in most patients as of POD 2. Most of them were not aware of the passage of flatus because the TDT placement resulted in the reduction of the endoluminal pressure. No differences were found between the two groups according to the postoperative complications (Table 2). The incidence of anastomotic leakage was similar (1%) in both groups, and these patients received an emergency ileostomy (Fig. 1). Postoperatively, one patient developed ileus because of a small intestinal stenosis at the site of the ileostomy. The mortality rate was zero in both groups. The median time until the start of diet intake was POD 5 (range 2–8) in the COF group and POD 2 (range 2–8) in the EOF group (*P* < 0.001). In the COF group, diet intake was started by POD 3 in 37 patients (39%), on POD 4–6 in 33 patients (35%), and on POD ≥7 in 25 patients (26%). All ten patients with a diverting ileostomy started diet intake on POD 2 or 3. Two patients undergoing a splenectomy with a distal pancreatectomy started diet intake on POD 4 and 6, respectively. On the other hand, in the EOF group, diet intake was started on POD 2 in 92 patients (87%), on POD 3–6 in 10 patients (9%), and on POD ≥7 in 4 patients (4%). Two of the four patients undergoing a splenectomy with a distal pancreatectomy started diet intake on POD 4, and the others started on POD 8. After oral feeding was started, the diet build-up sequence was acceptable for the patients in both COF and EOF groups except for those with anastomotic leakage or ileus at the site of the ileostomy. The median length of the postoperative hospital stay was 6 days shorter for the EOF group than for the COF group (Table 2). However, the duration from surgery until the start of postoperative chemotherapy was not significantly different in the two groups. Eight patients did not receive postoperative chemotherapy. The reasons for this were as follows: (1) 4 patients had stage IA (ovarian tumor was adherent to the rectum with endometriosis or inflammation) disease; (2) 2 patients had low-grade carcinoma; and (3) 2 patients refused postoperative therapy. Stoma closure was performed in 16 of the 18 patients with a diverting or emergency ileostomy at 1–3 months after the completion of the postoperative chemotherapy (Fig. 1). The remaining two patients were unable to undergo stoma closure because of disease regrowth while receiving postoperative chemotherapy consisting of a combination of paclitaxel and carboplatin.

## Discussion

A period of starvation after gastrointestinal surgery during which an intestinal anastomosis has been formed has been a common practice. The rationale for starvation was to prevent postoperative nausea and vomiting and to protect the anastomosis, allowing it time to heal before being stressed by food [11]. ERAS programs incorporate evidence-based practices to minimize perioperative stress, intestinal dysfunction, iatrogenic infections, and postoperative pain and to promote early mobilization and recovery. It has been shown that ERAS programs including EOF can significantly reduce the median postoperative stays of 2 to 3 days following colorectal surgery without an appreciable increase in complications in cohort and matched control studies [13–15]. In the field of gynecologic surgery, it has been demonstrated that implementation of as many ERAS elements as possible is associated with a decrease in the length of the hospital stay without increasing the postoperative complication rate or mortality [16, 22]. In a prospective controlled trial of a COF group (*n* = 72) versus an EOF group (*n* = 71) following major abdominal gynecologic surgery, the average length of stay for the COF group was 5.8 days, while that for the EOF group was 4.7 days (*P* = 0.006) [23]. In this study, 83 patients (58%) had ovarian cancer and 19 patients (13%) received upper abdominal surgery. The proportion of women who underwent rectosigmoid resection was not mentioned. In another prospective controlled trial comparing COF (*n* = 22) and EOF (*n* = 18) groups of gynecologic oncology patients undergoing intestinal resection, the average length of stay for the COF group was 9.1 days, while that for the EOF group was 6.9 days (*P* = 0.022) [16]. In this study, 30 patients (75%) underwent rectosigmoid resection and 15 patients (38%) received upper abdominal surgery. In these two prospective controlled trials, patients in the EOF group were initially given a clear liquid diet on POD 1. If well tolerated, they were then given a regular diet. In reviews of ERAS programs for major abdominal gynecologic surgery, EOF appeared to be safe without increased gastrointestinal morbidities or other postoperative complications. The benefits of this approach include a faster recovery of bowel function, lower rates of infectious complications, a shorter hospital stay, and higher patient satisfaction [17, 18, 24, 25].

The current study also demonstrated that EOF was feasible and safe for ovarian cancer patients undergoing rectosigmoid resection with anastomosis as a part of cytoreductive surgery. A long period of postoperative starvation seems to be unnecessary, even after high-complexity surgery. Consequently, EOF was associated with a shorter postoperative hospital stay. The lengths of the hospital stay were longer for both groups in the current series than those reported in previous studies. In our study, forty-seven patients (23%) underwent multiple bowel resections and 117 patients (58%) underwent upper abdominal surgery. Therefore, our operating time was much longer than the operating times in previously reported prospective controlled trials, which averaged about 3 h. The shortness of operative times might partly explain the intestinal function recovery immediately after cytoreductive surgery. Patients in the EOF group were given only liquids on POD 1 and a semisolid diet on POD 2. Our impression from the current study is that a diet of either clear liquids or semisolid food is not tolerable for most ovarian cancer patients undergoing high-complexity surgery, including both rectosigmoid resection and upper abdominal procedures, on POD 1. A proportion of advanced ovarian cancer patients can tolerate a diet on POD 1. We are planning a trial to investigate the safety and clinical usefulness of providing a postoperative diet on POD 1 for patients undergoing cytoreductive surgery with intermediate SCS. The medical insurance system in Japan fully covered the costs of their hospital stay regardless of the length. This system may influence the relatively longer hospital stays that are typical for patients in Japan. However, a comparison of the length of the hospital stay for patients undergoing rectosigmoid resection with anastomosis between the COF and EOF groups still showed a significant difference even after accounting for potential biases. This study did not evaluate patient satisfaction and quality of life in both COF and EOF groups. It has been reported that applying the ERAS to patients undergoing colorectal surgery can improve their satisfaction during hospitalization and quality of life after surgery as well as have economic advantages [26–28].

Some studies have shown that diverting stomas have the potential to decrease the frequency of anastomotic leakage in ovarian cancer patients undergoing rectosigmoid resection. Houvenaeghel et al. reported that 59 (20%) of 305 patients who underwent rectosigmoid resection with anastomosis during up-front surgery, interval debulking surgery, or secondary debulking surgery received a diverting stoma, and the overall anastomotic leakage rate was 8% in 9 French cancer centers [29]. Another study analyzed retrospective data from 331 patients with stages II–IV ovarian cancer who underwent colon resection during up-front surgery. Forty-four patients (13%) received a diverting ileostomy, and the overall anastomotic leakage rate was 6% [30]. In the current series, the incidence of anastomotic leakage was relatively low (1%) in both COF and EOF groups. However, it should be noted that the patient selection was limited. A diverting stoma was created for 8% of the patients who underwent rectosigmoid resection with anastomosis. The proportion of the patients who had a diverting stoma in the COF group was higher than that in the EOF group. Since the middle of this study period, we have routinely utilized a TDT after rectosigmoid resection with anastomosis for patients with ovarian cancer [20]. TDT placement can decrease the need for a diverting stoma after rectosigmoid resection with anastomosis. In both the COF and EOF groups, all patients with a diverting ileostomy started diet intake on POD 2 or 3.

Some limitations must be considered when interpreting the data from this study. This was a retrospective, not a randomized, study. From 2012 to 2016, most patients had COF except for the cases with a diverting ileostomy. From 2016 to 2019, most patients had EOF except for the cases who had undergone a splenectomy with a distal pancreatectomy or with severe postoperative complications. Though we performed rectosigmoid resection with anastomosis using the same surgical techniques, advances of skills and medical instrumentation might have occurred in the entire cytoreductive surgery in the later period. Despite these limitations, the surgery by the same gynecologic oncologists at a single institution, the consecutiveness of the patients, and the consistency of the surgical decisions should reduce the possibility of major biases.

## Conclusion

EOF provides a significant reduction in the length of the postoperative hospital stay without an increased risk of complications after rectosigmoid resection with anastomosis for the treatment of primary ovarian cancer.

## Data Availability

The datasets used and/or analyzed during the current study are available from the corresponding author upon reasonable request.

## References

[1] Du Bois A, Reuss A, Pujade-Lauraine E, Harter P, Ray-Coquard I, Pfisterer J (2009). Role of surgical outcome as prognostic factor in advanced epithelial ovarian cancer: a combined exploratory analysis of 3 prospectively randomized phase 3 multicenter trials: by the Arbeitsgemeinschaft Gynaekologische Onkologie Studiengruppe Ovarialkarzinom (AGO-OVAR) and the Groupe d’Investigateurs Nationaux Pour les Etudes des Cancers de l’Ovaire (GINECO). Cancer.

[2] Shih KK, Chi DS (2010). Maximal cytoreductive effort in epithelial ovarian cancer surgery. J Gynecol Oncol..

[3] Chang SJ, Bristow RE, Ryu HS (2012). Impact of complete cytoreduction leaving no gross residual disease associatedwith radical cytoreductive surgical procedures on survival in advanced ovarian cancer. Ann Surg Oncol.

[4] Eisenkop SM, Nalick RH, Teng NN (1991). Modified posterior exenteration for ovarian cancer. Obstet Gynecol.

[5] Bristow RE, del Carmen MG, Kaufman HS, Montz FJ (2003). Radical oophorectomy with primary stapled colorectal anastomosis for resection of locally advanced epithelial ovarian cancer. J Am Coll Surg.

[6] Kato K, Tate S, Nishikimi K, Shozu M (2013). Bladder function after modified posterior exenteration for primary gynecological cancer. Gynecol Oncol.

[7] Kato K, Nishikimi K, Tate S, Kiyokawa T, Shozu M (2015). Histopathologic tumor spreading in primary ovarian cancer with modified posterior exenteration. World J Surg Oncol.

[8] Richardson DL, Mariani A, Cliby WA (2006). Risk factors for anastomotic leak after recto-sigmoid resection for ovarian cancer. Gynecol Oncol.

[9] Son JH, Kim J, Shim J, Kong TW, Paek J, Chang SJ, Ryu HS (2019). Comparison of posterior rectal dissection techniques during rectosigmoid colon resection as part of cytoreductive surgery in patients with epithelial ovarian cancer: close rectal dissection versus total mesorectal excision. Gynecol Oncol.

[10] Aletti GD, Santillan A, Eisenhauer EL, Hu J, Aletti G, Podratz KC (2007). A new frontier for quality of care in gynecologic oncology surgery: multiinstitutional assessment of short-term outcomes for ovarian cancer using a risk-adjusted model. Gynecol Oncol.

[11] Lewis SJ, Egger M, Sylvester PA, Thomas S (2001). Early enteral feeding versus “Nil by Mouth” after gastrointestinal surgery: systematic review and meta-analysis of controlled trial. BMJ.

[12] Kehlet H (2008). Fast-track colorectal surgery. Lancet.

[13] Kehlet H, Mogensen T (1999). Hospital stay of 2 days after open sigmoidectomy with a multimodal rehabilitation programme. Br J Surg..

[14] Basse L, Hjort Jakobsen D, Billesbølle P, Werner M, Kehlet H (2000). A clinical pathway to accelerate recovery after colonic resection. Ann Surg.

[15] Basse L, Thorbøl JE, Løssl K, Kehlet H (2004). Colonic surgery with accelerated rehabilitation or conventional care. Dis Colon Rectum..

[16] Minig L, Biffi R, Zanagnolo V, Attanasio A, Beltrami C, Bocciolone L, Botteri E, Colombo N, Iodice S, Landoni F, Peiretti M, Roviglione G, Maggioni A (2009). Early oral versus “traditional” postoperative feeding in gynecologic oncology patients undergoing intestinal resection: a randomized controlled trial. Ann Surg Oncol.

[17] Nelson G, Altman AD, Nick A, Meyer LA, Ramirez PT, Achtari C, Antrobus J, Huang J, Scott M, Wijk L, Acheson N, Ljungqvist O, Dowdy SC (2016). Guidelines for pre- and intra-operative care in gynecologic/oncology surgery: enhanced recovery after surgery (ERAS®) society recommendations--part I. Gynecol Oncol.

[18] Nelson G, Altman AD, Nick A, Meyer LA, Ramirez PT, Achtari C, Antrobus J, Huang J, Scott M, Wijk L, Acheson N, Ljungqvist O, Dowdy SC (2016). Guidelines for postoperative care in gynecologic/oncology surgery: enhanced recovery after surgery (ERAS®) society recommendations--part II. Gynecol Oncol.

[19] Dindo D, Demartines N, Clavien PA (2004). Classification of surgical complications: a new proposal with evaluation in a cohort of 6336 patients and results of a survey. Ann Surg.

[20] Kato K, Omatsu K, Matoda M, Nomura H, Okamoto S, Kanao H, Utsugi K, Takeshima N (2018). Efficacy of transanal drainage tube placement after modified posterior pelvic exenteration for primary ovarian cancer. Int J Gynecol Cancer.

[21] Kato K, Tate S, Nishikimi K, Shozu M (2013). Management of pancreatic fistulas after a splenectomy as part of cytoreductive surgery for ovarian cancer. Int J Gynecol Cancer.

[22] Wijk L, Udumyan R, Pache B, Altman AD, Williams LL, Elias KM (2019). International validation of enhanced recovery after surgery society guidelines on enhanced recovery for gynecologic surgery. Am J Obstet Gynecol.

[23] Minig L, Biffi R, Zanagnolo V, Attanasio A, Beltrami C, Bocciolone L, Botteri E, Colombo N, Iodice S, Landoni F, Peiretti M, Roviglione G, Maggioni A (2009). Reduction of postoperative complication rate with the use of early oral feeding in gynecologic oncologic patients undergoing a major surgery: a randomized controlled trial. Ann Surg Oncol.

[24] Charoenkwan K, Matovinovic E (2014). Early versus delayed oral fluids and food for reducing complications after major abdominal gynaecologic surgery. Cochrane Database Syst Rev.

[25] Miralpeix E, Nick AM, Meyer LA, Cata J, Lasala J, Mena GE, Gottumukkala V, Iniesta-Donate M, Salvo G, Ramirez PT (2016). A call for new standard of care in perioperative gynecologic oncology practice: impact of enhanced recovery after surgery (ERAS) programs. Gynecol Oncol.

[26] Adamina M, Kehlet H, Tomlinson GA, Senagore AJ, Delaney CP (2011). Enhanced recovery pathways optimize health outcomes and resource utilization: a meta-analysis of randomized controlled trials in colorectal surgery. Surgery.

[27] Vlug MS, Wind J, Hollmann MW, Ubbink DT, Cense HA, Engel AF, Gerhards MF, van Wagensveld BA, van der Zaag ES, van Geloven AA, Sprangers MA, Cuesta MA, Bemelman WA, LAFA study group (2011). Laparoscopy in combination with fast track multimodal management is the best perioperative strategy in patients undergoing colonic surgery: a randomized clinical trial (LAFA-study). Ann Surg.

[28] Thiele RH, Rea KM, Turrentine FE, Friel CM, Hassinger TE, McMurry TL, Goudreau BJ, Umapathi BA, Kron IL, Sawyer RG, Hedrick TL (2015). Standardization of care: impact of an enhanced recovery protocol on length of stay, complications, and direct costs after colorectal surgery. J Am Coll Surg.

[29] Houvenaeghel G, Gutowski M, Buttarelli M, Cuisenier J, Narducci F, Dalle C, Ferron G, Morice P, Meeus P, Stockle E, Bannier M, Lambaudie E, Rouanet P, Fraisse J, Leblanc E, Dauplat J, Querleu D, Martel P, Castaigne D (2009). Modified posterior pelvic exenteration for ovarian cancer. Int J Gynecol Cancer.

[30] Tseng JH, Suidan RS, Zivanovic O, Gardner GJ, Sonoda Y, Levine DA, Abu-Rustum NR, Tew WP, Chi DS, Long Roche K (2016). Diverting ileostomy during primary debulking surgery for ovarian cancer: associated factors and postoperative outcomes. Gynecol Oncol.

